# *N*-acetylglucosamine-1-Phosphate Transferase Suppresses Lysosomal Hydrolases in Dysfunctional Osteoclasts: A Potential Mechanism for Vascular Calcification

**DOI:** 10.3390/jcdd2020031

**Published:** 2015-04-15

**Authors:** Yang Lei, Masaya Iwashita, Jung Choi, Masanori Aikawa, Elena Aikawa

**Affiliations:** Center for Interdisciplinary Cardiovascular Sciences, Division of Cardiovascular Medicine, Brigham and Women’s Hospital, Harvard Medical School, Boston, MA 02115, USA; E-Mails: ylei3@partners.org (Y.L.); miwashita@partners.org (M.I.); jchoi22@partners.org (J.C.); maikawa@rics.bwh.harvard.edu (M.A.)

**Keywords:** GNPTAB, vascular calcification, osteoclast, lysosomal hydrolases

## Abstract

In addition to increased differentiation of vascular smooth muscle cells into osteoblast-like phenotypes, the limited accumulation of osteoclasts in atherosclerotic plaques or their dysfunction may participate in potential mechanisms for vascular calcification. *N*-acetylglucosamine-1-phosphate transferase containing alpha and beta subunits (GNPTAB) is a transmembrane enzyme complex that mediates the vesicular transport of lysosomal hydrolases. GNPTAB may also regulate the biogenesis of lysosomal hydrolases from bone-marrow derived osteoclasts. In this study, the areas surrounding calcification in human atherosclerotic plaques contained high levels of GNPTAB and low levels of lysosomal hydrolases such as cathepsin K (CTSK) and tartrate-resistant acid phosphatase (TRAP), as demonstrated by immunohistochemistry and laser-capture microdissection-assisted mRNA expression analysis. We therefore hypothesized that GNPTAB secretion may suppress the release of CTSK and TRAP by vascular osteoclast-like cells, thus causing their dysfunction and reducing the resorption of calcification. We used human primary macrophages derived from peripheral blood mononuclear cells, an established osteoclast differentiation model. GNPTAB siRNA silencing accelerated the formation of functional osteoclasts as detected by increased secretion of CTSK and TRAP and increased their bone resorption activity as gauged by resorption pits assay. We concluded that high levels of GNPTAB inhibit secretion of lysosomal hydrolases in dysfunctional osteoclasts, thereby affecting their resorption potential in cardiovascular calcification.

## 1. Introduction

Previous studies have proposed several mechanisms for vascular calcification, including osteoblastic differentiation of smooth muscle cells (SMCs) [1,2,3], limited accumulation of osteoclasts and their dysfunction [4,5,6,7], loss of endogenous inhibitors of mineralization [8,9], cell death [10], and thermodynamic mechanisms via elevated calcium and phosphate levels [11]. Similarly, bone mineralization involves multiple mechanisms [12]. Bone homeostasis depends on the balance between bone formation by osteoblasts and bone resorption by osteoclasts [13]. Osteoclasts originated from hematopoietic progenitors of the monocyte/macrophage lineage differentiate into functional osteoclasts and resorb mineralized tissues [14]. To solubilize the mineral component of bone, osteoclasts secrete various enzymes such as tartrate-resistant acid phosphatase (TRAP) [15], cathepsin K (CTSK) [16], and carbonic anhydrase II [17]. TRAP, an osteoclast differentiation marker, regulates bone mineral matrix resorption [15]; CTSK cleaves collagen [16]; and carbonic anhydrase II facilitates proton production and thus maintains acidification of the resorption lacuna [17,18]. 

While the sources of osteoclasts and osteoblasts in vasculature and bone could be different, vascular calcification and bone mineralization share similar mechanisms, including the maintenance of a balance between bone forming osteoblasts and bone resorbing osteoclasts. Pluripotent cells exist in arteries, and studies showed that peripheral mononuclear phagocytic cells can differentiate into osteoclast-like cells with receptor activator of nuclear factor-κB ligand (RANKL) and macrophage colony-stimulating factor (MCSF) signaling [19]. Net calcium deposition in calcified atherosclerotic plaques occurs due to imbalance between the functional activity of osteoclast-like cells and osteoblast-like cells in vascular system [20,21]. 

*N*-acetylglucosamine-1-phosphate transferase, alpha and beta subunits (GNPTAB), is a transmembrane enzyme complex which catalyzes the formation of the mannose-6-phosphate (M6P) recognition on high mannose type oligosaccharides in lysosomal hydrolases. The M6P pathway regulates most lysosomal hydrolases, which are synthesized in the endoplasmic reticulum. The modified lysosomal hydrolases are then packaged into clathrin-coated transport vesicles for delivery to endosomes and lysosomes [22,23]. GNPTAB-deficient mice showed that osteoclasts derived from their bone marrow have increased secretion of TRAP and CTSK but not cathepsin D [24]. Another study used mucolipidosis type II-deficient mice, generated by a single-base insertion into the GNPTAB gene corresponding to a mutation detected in mucolipidosis type II patients [25], to show the increased formation of intact osteoclasts, combined with decreased activity of bone-forming osteoblasts [26]. The present study has tested the hypothesis that molecules commonly expressed by these two cell types such as GNPTAB serve as molecular switches differentially regulating osteoclast and osteoblast functions.

## 2. Materials and Methods

### 2.1. Human Carotid Arteries

Human carotid arteries (non-calcified arteries, *n* = 3; calcified atherosclerotic plaques, *n* = 6) were obtained from endarterectomy and autopsy specimens harvested following protocol approved by the Institutional Review Board of Brigham and Women’s Hospital (protocol # 1999P001348). Human tissues were analyzed by immunohistochemistry and immunofluorescence. Cryosections of 6-µm thickness were stained for GNPTAB, CD68, TRAP, and CTSK. Adjacent sections were double labeled for GNPATB and CD68 for colocalization analysis. Sections with high expression of GNPTAB in calcified plaques or no expression of GNPTAB in non-calcified regions were submitted for laser-capture microdissection (LMD 6500, Leica, Wetzlar, Germany). Calcified GNPTAB-enriched areas as well as non-calcified areas were captured in four adjacent 20-µm sections and pooled for RNA extraction using TRIzol reagent (Invitrogen, Carlsbad, CA, USA). 

### 2.2. Immunohistochemistry and Immunofluorescence for Human Tissues

Immunohistochemistry and immunofluorescence for human tissues were performed on fresh-frozen sections as previously described [27,28]. Tissue samples were frozen in OCT compound (Sakura Finetech, Torrance, CA, USA) and 6-µm sections were fixed in 4% paraformaldehyde, blocked in 4% horse serum and incubated with primary antibody for 90 min. Primary antibodies included GNPTAB (Novus Biologicals, Littleton, CO, USA), CD68 (Dako, Copenhagen, Denmark), TRAP (Abcam, Cambridge, MA, USA), and CTSK (Abcam, Cambridge, MA, USA). The avidin-biotin peroxidase method was used. The reactions were visualized with 3-amino-9-ethylcarbazole substrate (AEC substrate; Dako, Denmark). Sections treated with PBS without primary antibody were used as negative controls. The slides were scanned using 20× magnification for digital imaging on a GE Omnyx^TM^ VL4 scanner (Omnyx LLC, Pittsburgh, PA, USA) and exported from the Omnyx^TM^ Pathologist Work Station to view whole slide images. Areas of interest were also captured using 400× magnification with a charged-coupled device camera with a fixed aperture (Nikon DS-Fi1c, Nikon, Tokyo, Japan) on a Nikon ECLIPSE 50i microscope (Nikon, Tokyo, Japan). 

For immunofluorescence, GNPATB and CD68 were labeled with streptavidin-conjugated Alexa Fluor 594 antibody and streptavidin-conjugated Alexa Fluor 488 antibody, respectively. Sections were mounted with mounting medium containing 4',6-diamidino-2-phenylindole (DAPI, Vector Laboratories, Burlingame, CA, USA). Images were then captured using confocal microscopy (A1, Nikon). 

### 2.3. CD14-Immunoreactive Monocyte Isolation and Osteoclast Differentiation

CD14-immunoreactive monocytes were isolated from human peripheral blood mononuclear cells using Dynabeads^®^ FlowComp^TM^ Human CD14 Kit (Invitrogen, Carlsbad, CA, USA). Thirty milliliters of buffy coat (Research Blood Components, Boston, MA, USA) underwent lymphocyte separation using lymphocyte separation medium (LSM; MP Biomedicals, Santa Ana, CA, USA) and washed with Hank’s balanced salt solution (HBSS; Corning Cellgro, Manassas, VA, USA), specifically purified by CD14 antibody. This protocols yields up to 30–60 million CD14-immunopositive monocytes. 

These isolated CD14-immunoractive monocytes were maintained in alpha-MEM (Sigma, St Louis, MO, USA) supplemented with 10% fetal bovine serum (FBS; Life Technologies, Rockville, MD, USA), 1% l-glutamine, penicillin, and streptomycin (Sigma) and cultured on 6-well plate (2 × 10^6^ cells/well) for mRNA and protein isolation, Corning Osteo Assay Surface 24-well plate for bone resorption analysis (0.5 × 10^6^ cells/well; Corning Life Sciences, Lowell, MA), and 96-well plate for TRAP staining and immunofluorescence. 

CD14-immunoractive monocytes underwent three following treatments: (1) macrophage colony-stimulating factor (MCSF, 25 ng/mL; PeproTech, Rocky Hill, NJ, USA) for macrophage control; (2) MCSF (25 ng/mL) + soluble human receptor activator of nuclear factor-κB ligand (RANKL, 30 ng/mL; PeproTech, Rocky Hill, NJ, USA) with non-targeting (NT) scrambled control siRNA (NT siRNA; SMARTpool siGENOME siRNA; GE Healthcare, Lafayette, CO, USA); and (3) MCSF followed by treatments with RANKL and GNPTAB siRNA (siGNPTAB; SMARTpool siGENOME siRNA; GE Healthcare, CO, USA). Group 1 served as negative control in which cells were maintained as macrophages; group 2 served as positive control in which cells were differentiated into osteoclasts, and group 3 represented differentiated osteoclasts treated with siRNA GNPTAB. The medium was changed twice a week and cells were maintained for 14 days.

### 2.4. siRNA Transfection Experiments

Small interfering RNA (siRNA) transfer to human CD14-immunoreactive monocytes used SilenceMag (Boca Scientific, Boca Raton, FL, USA). Fifty nanometers of siRNA was diluted in reduced-serum Opti-MEM^®^ Medium (Life Technologies, Gaithersburg, MD, USA), mixed with the SilenceMag reagent, and incubated at RT. This mixture was added drop by drop onto the cells. Cell cultures were placed on the magnetic plates for 15 min. After 6 h, medium was changed. The siRNA transfection procedures were performed on day 4, day 7, and day 10. By the end of the experiment on day 14, efficiency of the knockdown was determined at both mRNA and protein levels. Cells were lysed using TRIzol reagent (Invitrogen, Carlsbad, CA, USA) and analyzed by RT-PCR. The protein expression was assessed by immunofluorescence. 

### 2.5. Real-Time PCR Analysis

Total RNA from both human tissue and human CD14-immunoreactive monocytes were isolated by TRIzol reagent (Invitrogen, Carlsbad, CA, USA) and reversed transcribed using qScript^TM^ cDNA Synthesis Kit (Quanta Biosciences, Gaithersberg, MD, USA). Real-time quantitative PCR was performed using Taqman^®^ Gene Expression Assay probes (Applied Biosystems, Foster City, CA, USA; Supplemental Table S1) TaqMan^®^ fast universal master kit (Applied Biosystems, Foster City, CA, USA) by Applied Biosystems^®^ 7900HT Fast Real-Time PCR System and normalized by GAPDH. Relative fold changes were calculated by the 2^−ΔΔCT^ method. 

### 2.6. Immunofluorescence for Human Cells

Human CD14-immunoreactive monocytes were washed with PBS, fixed by 4% formaldehyde, blocked with 2% bovine serum albumin (BSA) and 0.05% Tween-20 in PBS. For silencing efficiency of GNPTAB, cells were incubated with rabbit anti-GNPTAB (Novus Biologicals, Littleton, CO, USA) for 90 minutes at room temperature, followed with AlexaFlour^®^ 488-conjugated anti-rabbit IgG antibody (Life Technologies, Gaithersburg, MD, USA) for 60 min at room temperature and then processed for imaging. For cellular morphology study, cells were incubated with Alexa Fluor^®^ 594 phalloidin (Life Technologies, Gaithersburg, MD, USA) for 30 min at room temperature. Cells were washed three times with PBS and mounted with mounting medium containing DAPI (Vector Laboratories). Images were captured with confocal microscope A1 (Nikon, Tokyo, Japan). 

### 2.7. TRAP Staining and Quantification

TRAP staining and quantification used the TRAP staining kit (B-Bridge International, Sunnyvale, CA, USA). Cells on 96-well plates were washed by PBS, fixed by 10% formalin for 5 min at room temperature, stained by chromogenic substrate for up to 60 min until the best color condition was obtained. Images were taken After TRAP staining by light microscope and used to count the number of osteoclast-like cells. Detailed method is provided in online only Supplemental Materials (Figure S1). Briefly, TRAP-positive cells that had at least three nuclei were counted as differentiated osteoclasts [29,30] (Figure S1A). Five random-chosen areas were used to count osteoclast numbers followed by statistical analysis (Figure S1B). For quantification of TRAP activity, cell lysates or supernatant media were incubated with by chromogenic substrate for 3 h at 37 °C and then read in a microplate reader at 540 nm.

### 2.8. Resorption Assay for Osteoclast Functional Analysis

By the end of experiments (day 14), cells were removed with 10% bleach for 10 min at room temperature. The wells were washed twice with distilled water, dried for 3–5 h at room temperature and then resorption pit images were taken using a light microscope (Nikon ECLIPSE 50i microscope; Nikon, Tokyo, Japan). Images were converted to black and white binary images for quantification of pit resorption area using the image J [31]. 

### 2.9. CTSK Activity Assay

CTSK activity was measured using Cathepsin K activity assay kit (Abcam), a fluorescence-based assay that utilizes the preferred cathepsin-L substrate sequence LR labeled with AFC (amino-4-trifluoromethyl coumarin). The released AFC can easily be quantified using a fluorescence plate reader. Ten microliters of cell lysates or 50 µL of supernatant media were added with 50 µL of cathepsin K reaction buffer and 2 µL of the 10 mM Ac-LR-AFC substrate, incubated at 37 °C for 1–2 h then read in a microplate reader (400 nm excitation/505 nm emission).

### 2.10. Statistics

Results are expressed as means ± standard error of the mean (SEM). Statistical analyses used single-factor ANOVA. Data were considered significant with *p* value <0.05. Asterisks in figures denote statistical significance (*p* < 0.05) for each group compared with controls. 

## 3. Results

### 3.1. Human Calcified Arteries Contain Enriched GNPTAB around Calcified Areas but Low Levels of Osteoclast Lysosomal Hydrolases

**Figure 1:**
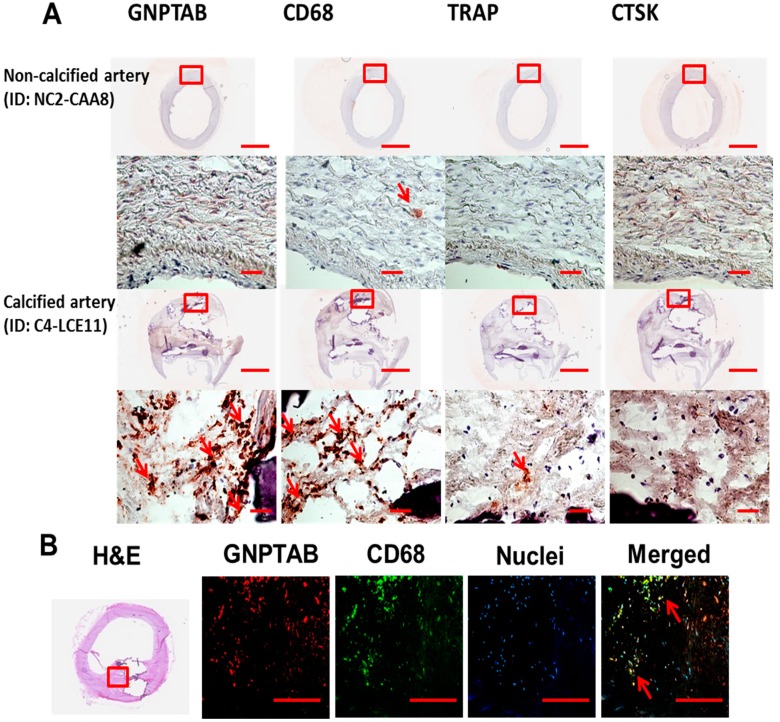
(**A**) Representative non-calcified and calcified arteries showed high expression of GNPTAB around and within CD68-immunopositive macrophages (red reaction product) but low expression of TRAP and CTSK in calcified arteries. No GNPTAB expression was observed in non-calcified arteries. Top row: bar = 2 mm; bottom row: bar = 500 µm. *n* = 3: non-calcified arteries; *n* = 6: calcified arteries; (**B**) Double fluorescence labeling for GNPTAB and CD68 identified a large population of macrophages co-expressing GNPTAB (arrows). Bars = 200 µm.

Regions around calcified areas of human carotid arteries contained many macrophages, detected by CD68, and prominent expression of GNPTAB (Table 1, Figure 1A and online only Supplemental Figure S2). The evidence has suggested the discrepancy between limited numbers of osteoclasts and abundance of macrophages, precursors of osteoclasts, in calcifying atherosclerotic plaques. The reason behind the lack of fully differentiated multinuclear cells that are able to resorb calcium deposition in cardiovascular tissues is unknown. Our histopathological observations showed that two major osteoclast lysosomal hydrolases, TRAP and CTSK, had negligible expression within GNPTAB-immunoreactive cells, surrounding calcifying regions, indicating that GNPTAB may affect TRAP and CTSK expression and thus suppress osteoclast function (Table 1, Figure 1A and online only Supplemental Figure S2). Double labeling for GNPTAB and CD68 showed that large populations of macrophages express GNPTAB (Figure 1B). 

**Table 1:** An analysis for immunohistochemistry (IHC) studies including GNPTAB, CD68, TRAP, and CTSK expression in non-calcified (*n* = 3) and calcified (*n* = 6) human arteries. Our quantitative criteria for relative abundance of expression include “-” for 0% of positive cells; “+” for ≤25%; “++” for ≤50%; “+++” for ≤75%; and “++++” for ≤100%. C: calcified; NC: non-calcified; CAA: carotid artery from autopsy specimens; LCE: left carotid artery from endarterectomy specimens; RCE: right carotid artery from endarterectomy specimens.

Human Arteries ID	GNPTAB	CD68	TRAP		CTSK
**Non-Calcified Human Carotid Arteries (*n* = 3)**					
NC1-CAA5	-	+	-	+	
NC2-CAA8	-	+	-	+	
NC3-CAA11	-	+	-	+	
**Calcified Human Carotid Arteries (*n* = 6)**					
C1-CAA2b	+++	++++	-	+	
C2-LCE1	++	++++	+	+	
C3-LCE4	++++	++++	+	-	
C4-LCE11	++++	++++	+	-	
C5-RCE7	+	++	-	-	
C6-RCE10	++	++++	+	-	

**Figure 2:**
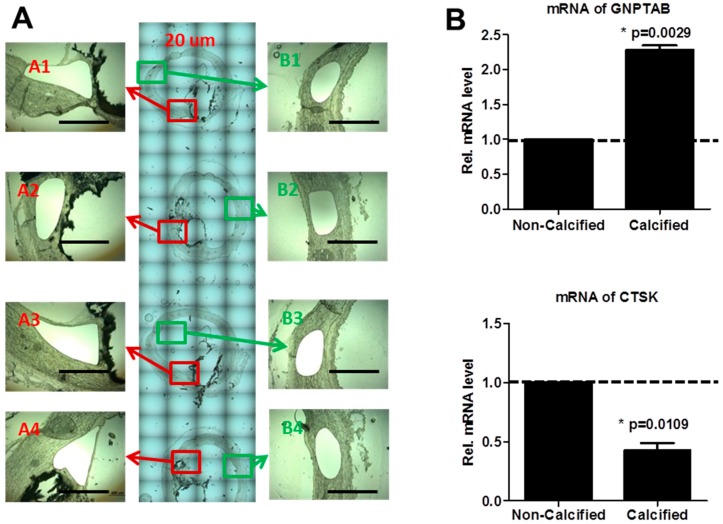
(**A**) Laser-capture microdissection-assisted mRNA analysis in human calcified carotid arteries. Four adjacent 20-µm sections both from GNPTAB-positive calcified areas (A1–A4) and GNPTAB-negative non-calcified areas (B1–B4) were pooled for RNA extraction. Bars = 500 µm; (**B**) mRNA levels of GNPTAB and CTSK in GNPTAB-positive calcified areas *versus* GNPTAB-negative non-calcified areas. (*n* = 4; two technical replicates each).

Laser-capture microdissection (LCM) allows rapid one-step collection from a section of complex and heterogeneous tissue [32], which is then can be used to extract DNA, RNA and proteins [33]. To further examine gene expression, we used this method to dissect GNPTAB-immunoreactive cells adjacent to calcified areas and GNPTAB-negative regions within non-calcified areas (Figure 2A). Calcified areas had significant increase of GNPTAB mRNA level *versus* non-calcified areas (Figure 2B; 2.28 ± 0.10 fold change, *p* = 0.0029, *n* = 4; two technical replicates) accompanied by a decrease of CTSK mRNA levels (Figure 2B; 0.42 ± 0.09 fold change, *p* = 0.0109, *n* = 4; two technical replicates). Of note, TRAP mRNA levels were non-detectable in both calcified and non-calcified areas. These results indicate that GNPTAB expression around calcified regions inhibit secretion of lysosomal hydrolases such as TRAP and CTSK, which can cause the dysfunction of osteoclast-like cells in atherosclerotic plaques. 

### 3.2. GNPTAB Silencing Increases Osteoclastogenesis and Resorption Pits Formed by Functional Osteoclast-Like Cells

**Figure 3:**
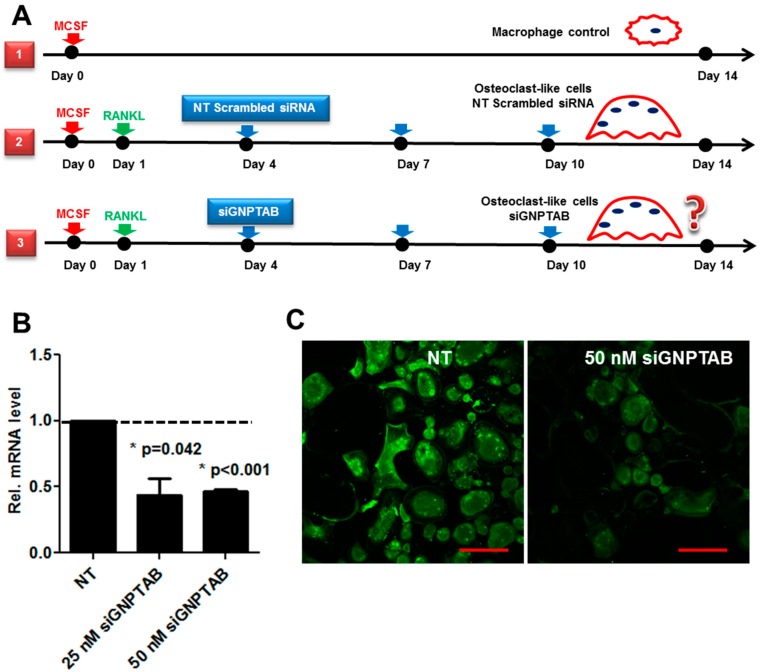
(**A**) Schematic illustrates the treatment protocol for isolated CD14-immunoreactive monocytes. Group 1, control group; group 2, differentiated osteoclasts treated with non-targeting (NT) scrambled control siRNA; and group 3, differentiated osteoclasts treated with siRNA GNPTAB; (**B**,**C**) Silencing efficiency was determined at both mRNA and protein levels by immunofluorescence. Bars = 200 µm (*n* = 4; two technical replicates each).

To validate our proposed mechanisms on the role of GNPTAB in osteoclast differentiation, we used human peripheral blood mononuclear cells (PBMC), an established model for osteoclast differentiation [34]. The experimental design is shown in the schematic (Figure 3A). We used the following groups: (1) MCSF treatment group, a negative control for non-differentiated CD14-immunoreactive monocytes/macrophages; (2) MCSF, RANKL, and non-targeting (NT) scrambled control siRNA treatments group to induce osteoclast differentiation; (3) MCSF, RANKL and siRNA GNPTAB treatments group to test the effects GNPTAB on osteoclastogenesis and osteoclast bone resorption function. Efficiency of the knockdown experiments was determined at both mRNA and protein levels by immunofluorescence (Figure 3B,C). The concentration of 50 nM siRNA for GNPTAB showed sufficient silencing efficiency and therefore was used in all of the following experiments.

**Figure 4:**
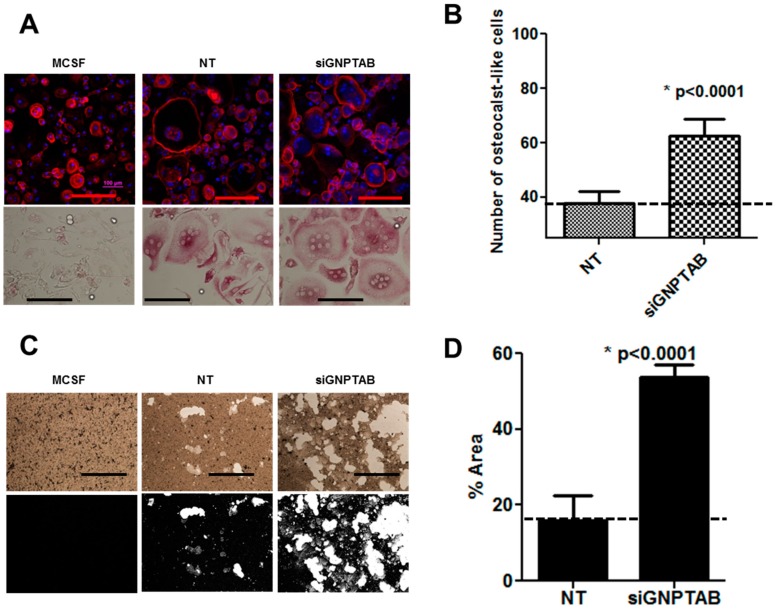
(**A**) Cellular morphology by F-actin immunofluorescence (top row) and TRAP staining (bottom row). F-actin rings were stained by Alexa Fluor^®^ 594 phalloidin (Red) and Nuclei were stained by DAPI (Blue). TRAP activity assay showed increased numbers of multinucleated osteoclast-like cells. Top row: bars = 200 µm. Bottom row: bars = 200 µm. (*n* = 4, two or five technical replicates each); (**B**) Number of osteoclast-like cells counted based on TRAP staining (*n* = 4, two or five technical replicates each); (**C**) Pit resorption assays showed resorption pits (white color) formed by functional osteoclasts (*n* = 4, one or two technical replicates each). Black and white binary images converted by Image J. Bars = 50 µm; (**D**) Percentages of pit areas quantified by Image J (*n* = 4, one or two technical replicates each).

Our results showed that osteoclasts treated with siRNA GNPTAB were smaller in size and more numerous as compared to NT scrambled control siRNA treatments (Figure 4A). The number of multinucleated TRAP-positive osteoclasts increased in the siRNA GNPTAB group (Figure 4B; 62.61 ± 6.03 *versus* 37.64 ± 4.566, *p* < 0.0001; See online Supplemental Materials for raw images: Figure S3A). To demonstrate the functional activity of osteoclasts, untreated cells, NT scrambled control siRNA treated osteoclasts and siRNA GNPTAB-treated cells were grown on an inorganic crystalline calcium phosphate plates designed to mimic *in vivo* bone environment [35]. We found a significant increase in the areas of resorption pits formed by osteoclasts in the siRNA GNPTAB group as compared to NT scrambled control siRNA treated group (Figure 4C,D; 53.80 ± 3.11 *versus* 16.03 ± 6.59, *p* < 0.0001; See online Supplemental Materials for raw images: Figure S3B), suggesting that CNPTAB silencing increases osteoclast differentiation and functionality. 

### 3.3. GNPTAB Silencing Increased Secretion of Lysosomal Hydrolases

**Figure 5:**
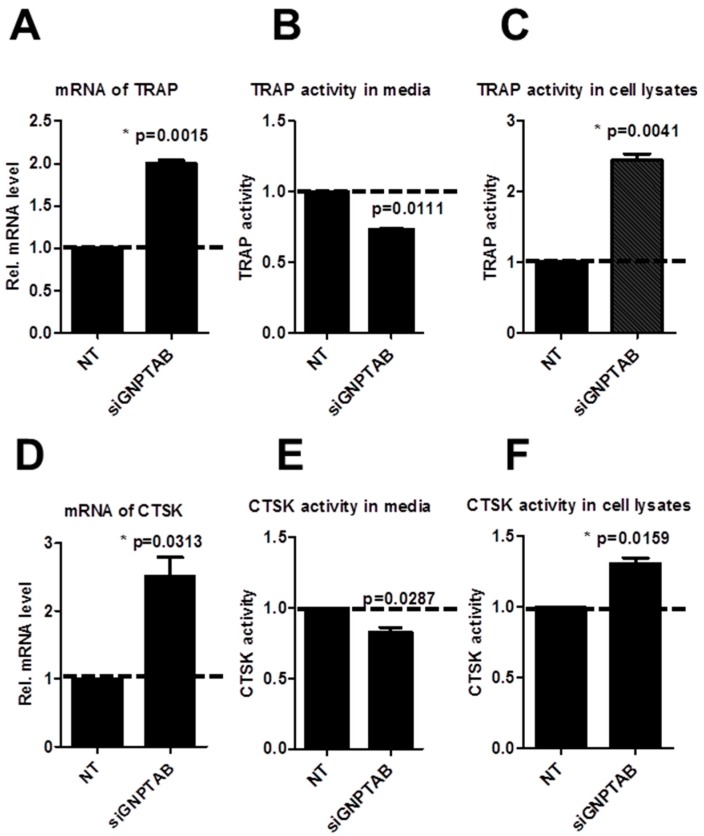
(**A**–**C**) mRNA levels of TRAP and TRAP activity in supernatant media and cell lysates (*n* = 2; two technical replicates each); **(D**–**F**) mRNA levels of CTSK and CTSK activity in supernatant media and cell lysates (*n* = 2; two technical replicates each).

To further elucidate the mechanisms of GNPTAB effects on osteoclastogenesis, we evaluated two major osteoclast lysosomal hydrolases, TRAP and CTSK, both at the mRNA and protein levels. The expression of both TRAP and CTSK increased at mRNA levels (2.00 ± 0.06, *p* = 0.0015; 2.51 ± 0.39, *p* = 0.0313-fold change; Figure 5A,D) and protein levels in cell lysates (2.44 ± 0.13, *p* = 0.0041; 1.31 ± 0.06, *p* = 0.0159-fold change; Figure 5C,F). We did not find changes in TRAP and CTSK mRNA levels in supernatant media (Figure 5B,E), suggesting that the secreted lysosomal hydrolases could be trapped in a sealed space formed by osteoclasts [14]. 

## 4. Discussion

The findings of this study have provided evidence for the role of GNPTAB in vascular calcification. We found that human calcified atherosclerotic plaques contain higher protein and mRNA levels of GNPTAB around calcified areas than non-calcified plaques, associated with low expression of osteoclast lysosomal hydrolases. Based on previous studies and our own findings, we propose a link between GNPTAB, osteoclast lysosomal hydrolases, osteoclast functional activity, and vascular calcification in atherosclerotic plaques. Our experiments demonstrated that high expression of GNPTAB associates with a low CTSK and TRAP expression in calcified arteries, leading to our central hypothesis that GNPTAB inhibits secretion of lysosomal hydrolases and causes osteoclast dysfunction, which negatively affects resorption of calcium deposition in the arterial wall. Our *in vitro* loss-of-function mechanistic evidence that GNPTAB silencing by siRNA increases expression of lysosomal hydrolases and improves osteoclast function supports our hypothesis. 

Numerous studies have investigated the osteoclast biology in skeletal system, but studies exploring the mechanisms for osteoclast dysfunctions in the vascular system are few. Multinuclear TRAP- and CSTK-positive osteoclast-like cells exist in calcified vascular tissues [4]. However, why osteoclast-like cells are sporadic in cardiovascular tissues and whether they have reduced ability to reverse calcification in calcified arteries remain unknown. The limited life span after implantation have reduced the effects of osteoclast-mediated therapies [5,36]. Studies also showed mechanisms of osteoclast dysfunction in osteopetrosis patients and found abnormal osteoclastogenesis and lack of osteoclast-specific adhesion structures [7]. Incomplete understanding of osteoclastogenesis in cardiovascular tissues has driven us to explore the specific molecules that can regulate osteoclast differentiation in the vasculature.

**Figure 6:**
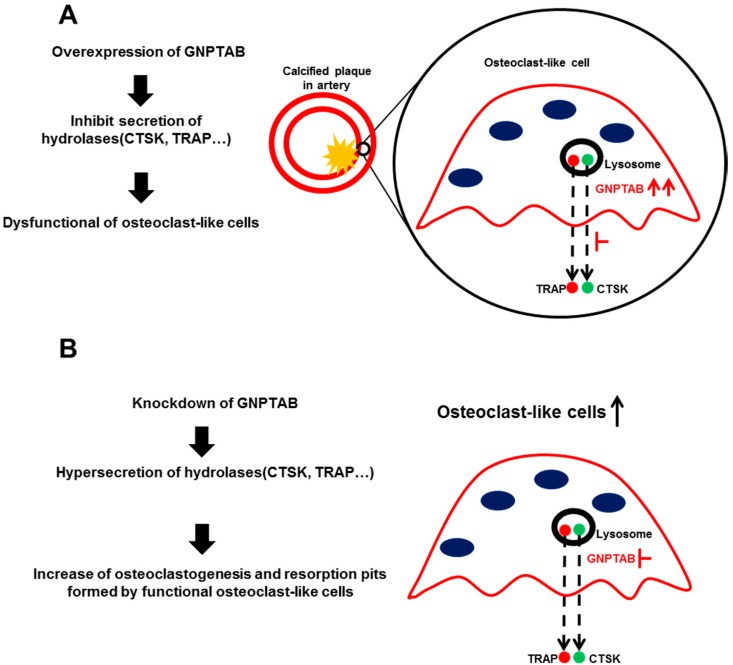
Proposed mechanisms of GNPTAB on regulating of osteoclast function. (**A**) High levels of GNPTAB around calcified areas in atherosclerotic plaques inhibit secretion of lysosomal hydrolases such as CTSK and TRAP, which causes dysfunction of osteoclast-like cells followed by lack of resolution of vascular calcification; **(B**) Silencing of GNPTAB enhances secretion of lysosomal hydrolases, resulting in improved osteoclast function.

This study proposed the mechanism for GNPTAB-induced osteoclast dysfunction in vascular calcification (Figure 6). Our initial observations in human calcified carotid arteries demonstrated high expression of GNPTAB around calcified regions but low in non-calcified arteries. In osteoclast biology, many critical molecules control osteoclast differentiation and function. Two acid hydrolases highly expressed and secreted by differentiated osteoclasts are CTSK and TRAP. CTSK degrades type I collagen and osteonectin, two protein components of bone matrix [37]. TRAP is a metallophosphoesterase involved in bone matrix degradation. TRAP reacts with hydrogen peroxide to produce highly destructive reactive oxygen species (ROS) that can destroy collagen [38]. TRAP also participates in removing the M6P recognition receptor from lysosomal proteins [39]. GNPTAB modifies the M6P residues of CTSK and TRAP, allowing their recognition by M6P receptors in the Golgi apparatus and ensuring their transport to lysosomal system [23]. Disruption of the M6P pathway in GNPTAB-deficient osteoclasts from bone marrow-derived macrophages leads to dysregulated secretion of CTSK and TRAP [24]. 

To better model the physiological environment in the vascular system, we employed PBMC-derived macrophages stimulated with MCSF and RANKL, commonly used for osteoclast differentiation [34]. *In vitro* differentiation of human primary monocytes into mature macrophages requires MCSF [14]. Macrophage differentiation into osteoclasts commonly uses RANKL, a type 2 membrane protein of the tumor necrosis factor superfamily [40]. Previous studies showed that GNPTAB regulates osteoclastogenesis of bone marrow-derived cells [24]. Our findings supported similar mechanisms for the role of GNPTAB in regulating CTSK and TRAP in blood-derived osteoclasts. Silencing of GNPTAB by siRNA increased mRNA expression levels of lysosomal hydrolases in cell lysates but not in supernatant, indicating the possibility that these lysosomal hydrolases are trapped within a sealed space formed by ruffled border of osteoclasts or in intracellular space. Our findings agree with previously published results suggesting that bone matrix degradation occurs not only extracellularly in the resorption lacunae but also intracellularly in the transcytotic vesicles [38]. In GNPATB-deficient mice, GNPTAB increased osteoclastogenesis and decreased osteoblastogenesis [26]. However, we did not find significant changes in human SMC-derived osteoblast-like cells. Silencing of GNPTAB in human SMC exerted no effects on calcium deposition and tissue non-specific alkaline phosphatase (TNAP) activity (Online Supplemental Figure S4). GNPTAB silencing in human SMC did not affect their osteoblast differentiation, suggesting that the previously proposed role of GNPTAB in mouse osteoblasts may not represent the same mechanisms in humans. 

Within the scope of the authors’ knowledge, we for the first time reported enriched GNPATB around calcified arteries. The question as to how GNPTAB is upregulated in calcified area requires more investigation. We propose that in calcified atherosclerotic plaques, circulating monocytes/macrophages accumulate around the calcified areas and secrete lysosomal hydrolyses capable of degrading minerals, mainly hydroxyapatite. Since the majority of soluble acid hydrolases are modified by the M6P pathway, this triggers up-regulation of GNPTAB, which is one of the major functional components of the M6P pathway [22]. 

Taken together, our studies suggest that GNPTAB does not regulate both osteoclast and osteoblast functions in humans, while exhibiting this function in mice. However, this evidence supports the therapeutic potential of GNPTAB inhibition to promote calcification resorption in human calcified atherosclerotic plaques by improving osteoclast function without affecting SMC osteogenic potential. Since GNPTAB regulates the majority of more than 60 lysosomal enzymes besides CTSK and TRAP [22], more studies are needed to investigate the role of GNPTAB in both vascular and bone tissues. 

## 5. Conclusions

We linked the GNPTAB to vascular calcification. Our GNPTAB siRNA silencing experiments supported the proposed mechanisms that GNPTAB regulates osteoclast function via increased CATK and TRAP production, facilitating osteoclast differentiation and improving their resorption function. In contrast, high expression levels of GNPTAB may delay macrophage differentiation into functional osteoclast-like cells characterized by low secretion levels of lysosomal hydrolases, which suppresses calcium deposition resorption in atherosclerotic plaques. Further investigation of GNPTAB and M6P pathway in vascular system may lead to the development of novel therapies for cardiovascular calcification. 

## Supplementary Materials

Supplementary materials can be accessed at: http://www.mdpi.com/2308-3425/2/2/0031/s1.
