# Supplementary material for: N-acetylglucosamine-1-Phosphate Transferase Suppresses Lysosomal Hydrolases in Dysfunctional Osteoclasts: A Potential Mechanism for Vascular Calcification
Source: J Cardiovasc Dev Dis. 2015 Apr 15;2(2):31–47. doi: 10.3390/jcdd2020031 (PMC5753093; doi:10.3390/jcdd2020031)
Supplement: Supplementary file 1 [file jcdd-02-00031-s001.pdf]

Supplemental Materials

Table S1. Taqman® probes used for real time PCR.

Gene Symbol	Assay ID	Gene Name
GAPDH	Hs02758991_g1	glyceraldehyde-3-phosphate dehydrogenase
GNPTAB	Hs00225647_m1	*N*-acetylglucosamine-1-phosphate transferase, alpha and beta subunit
CTSK	Hs00166156_m1	cathepsin K
ACP5	Hs00356261_m1	acid phosphatase 5, tartrate resistant; TRAP (Gene Aliases)

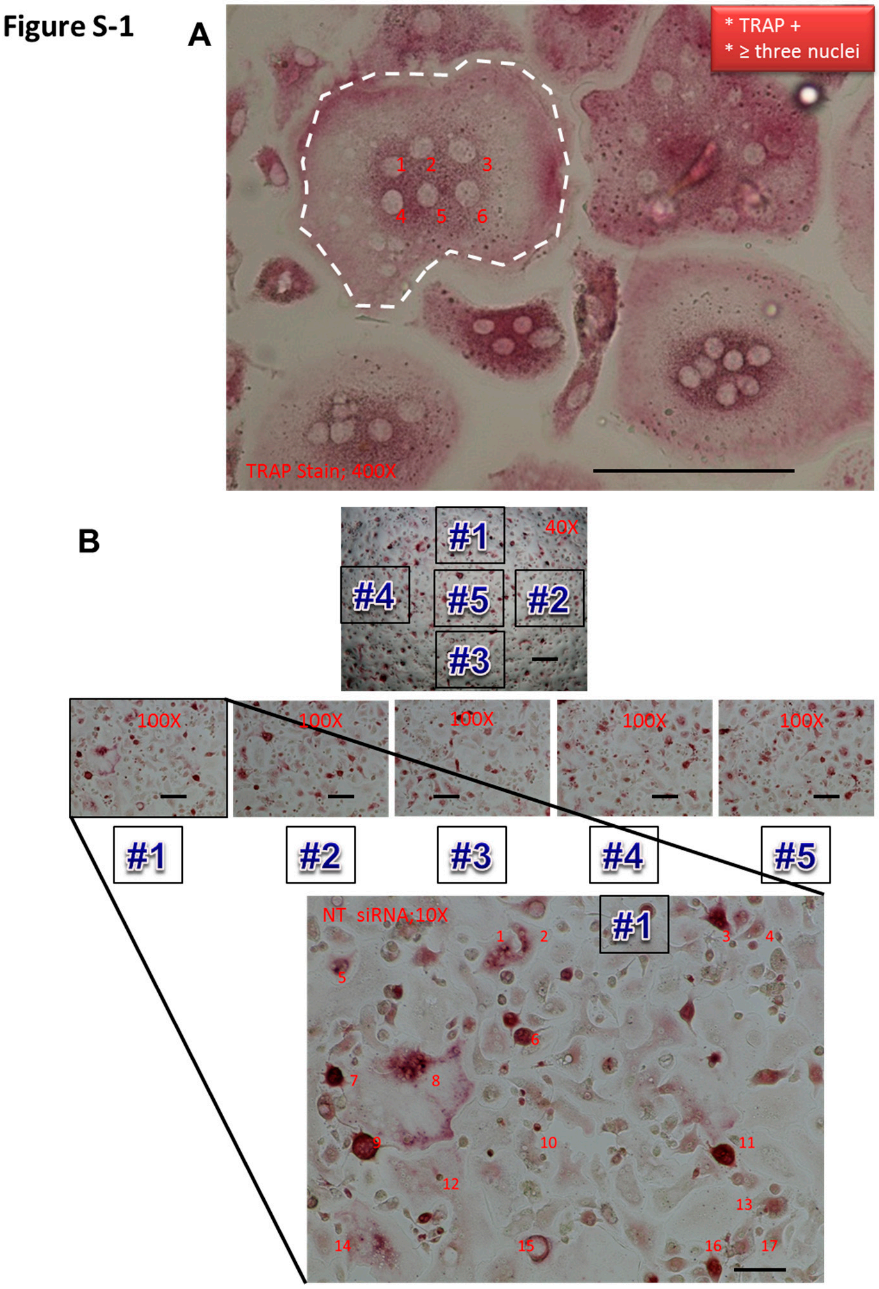

Figure S1. (**A**) A representative TRAP staining at high magnification (400X). TRAP-positive cells with at least three nuclei were counted as osteoclasts. For example, TRAP-positive cells traced by white dashed line had six nuclei and, therefore, have been counted as an osteoclast. Bars = 200 µm; **(B)** Five random chosen areas were used to count osteoclasts followed by statistical analysis (100× magnification). Bars = 400 µm.

Figure S-2A

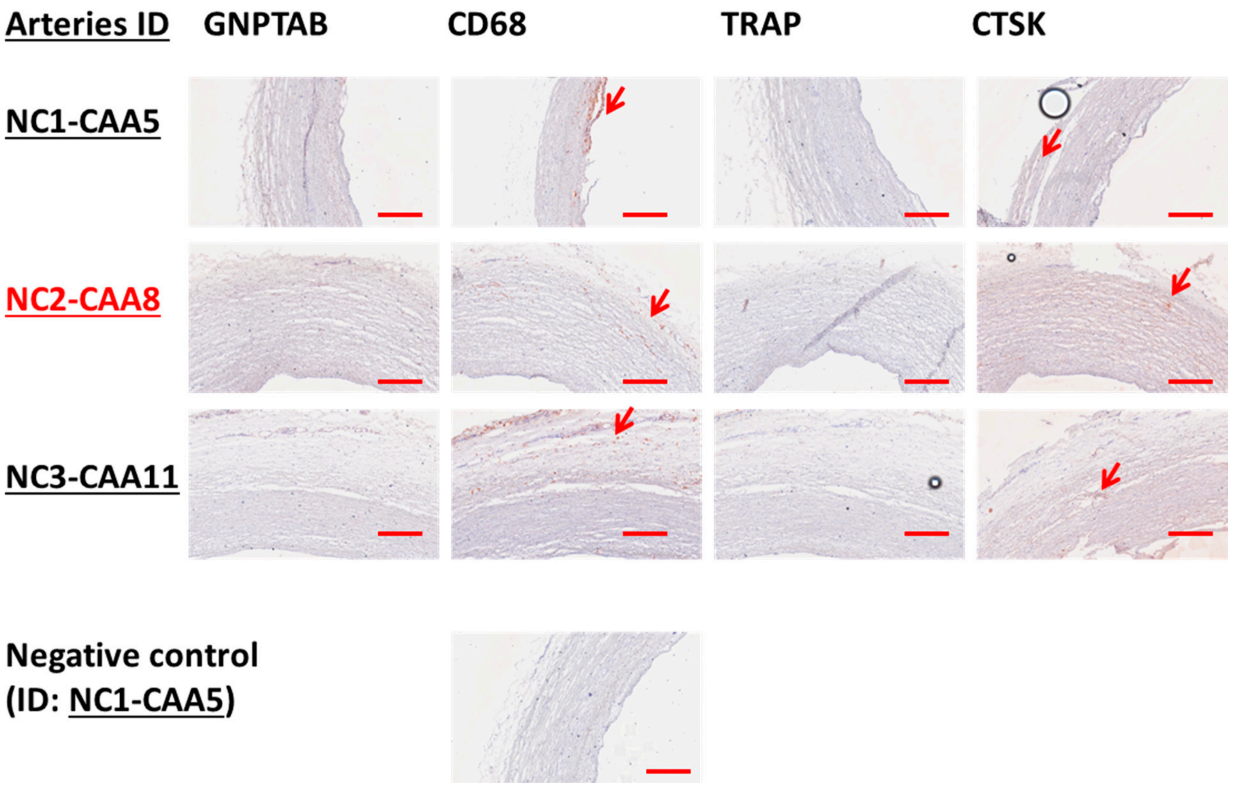

Figure S-2B

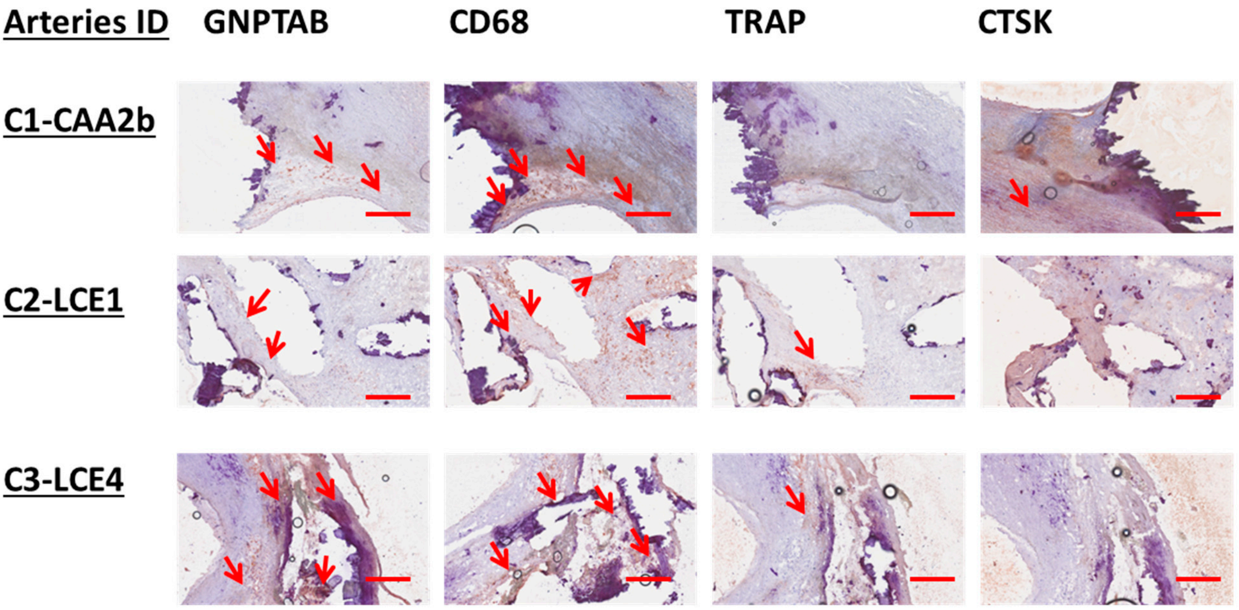

Figure S2. *Cont.*

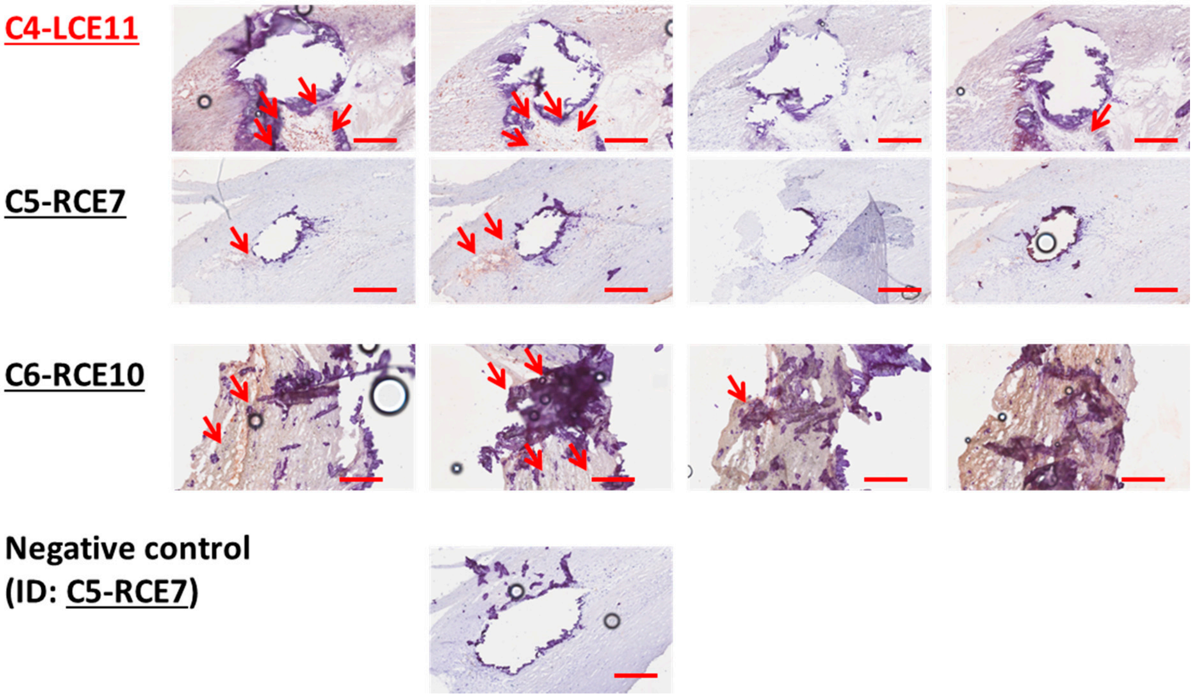

Figure S2. Representative immunohistochemistry images for GNPTAB, CD68, TRAP, and CTSK expression in non-calcified (*n* = 3) and calcified (*n* = 6) human arteries. Panel **A**: Non-calcified arteries; Panel **B**: Calcified arteries. ID: C: calcified; NC: non-calcified; CAA: carotid artery from autopsy specimens; LCE: left carotid artery from endarterectomy specimens; RCE: right carotid artery from endarterectomy specimens. Bar = 200 µm

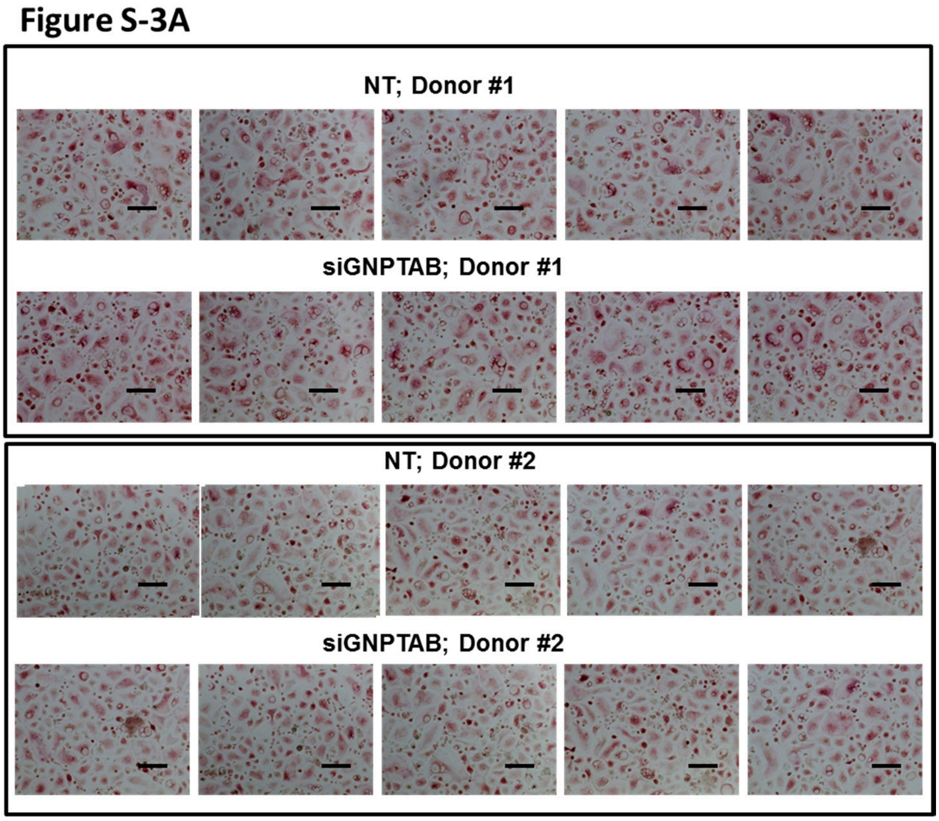

Figure S3. *Cont.*

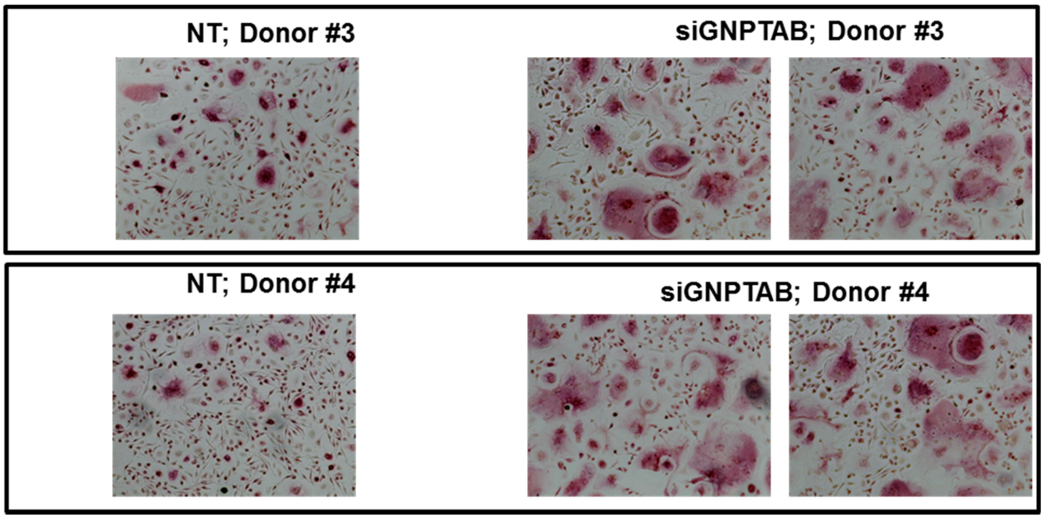

Figure S-3B

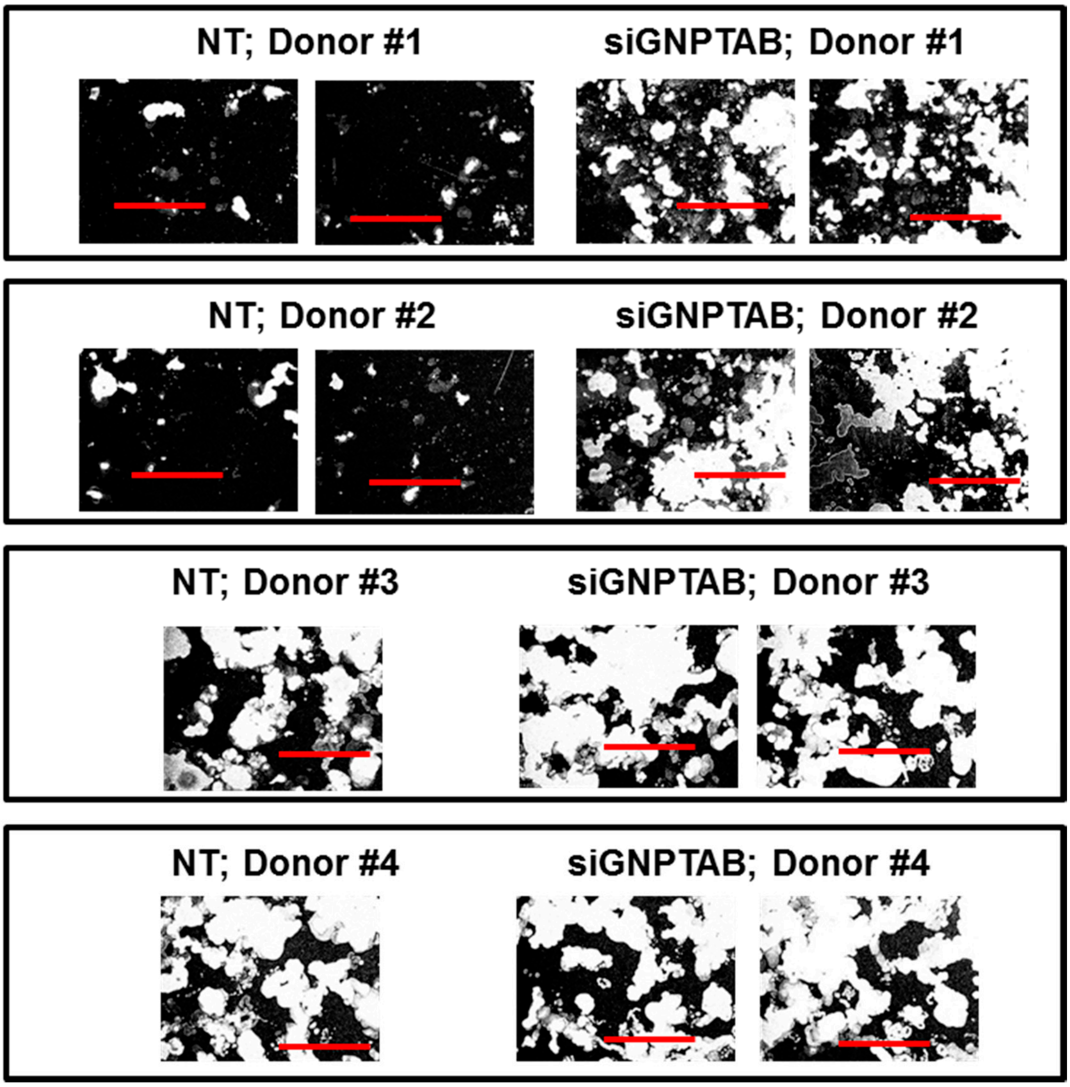

Figure S3. (**A**) Original images for TRAP staining from four donors. Bars = 400 µm. $n = 4$; two or five technical replicates each; **(B)** Original images for pit resorption areas from four donors. Bars = 50 µm. $n = 4$; one or two technical replicates each.

Material and Methods for Osteoblast Differentiation Experiments

(1) Cell culture

Human coronary artery smooth muscle cells (HCASMCs) were maintained in Smooth Muscle Cell Growth Medium 2 (Promocell), and were seeded in 48 well plates at 0.35×10^5 cells/well or 24 well plates at 0.75×10^5 cells/well. The next day, medium was changed to normal medium (NM) which was based on 10% FCS DMEM (Sigma) with 50 nM siRNAs using DharmaFECT 1 Transfection Reagent (GE Healthcare). After 3 days, the medium was changed to osteogenic medium (OM) which include 10 nM dexamethasone (Sigma), 10 mM β-glycerophosphate (Calbiochem) and 100 µM ascorbic acid 2-phosphate (Sigma) (It's based on 10% FCS DMEM (Sigma)) with siRNAs. The efficiency of the knockdown was determined at the same time (Day0). The medium with siRNAs was changed each 3–4 days for 21 days.

(2) Staining

For tissue non-specific alkaline phosphatase (TNAP) staining, HCASMCs were washed twice with PBS and stained with working BCIP/NBT Solution (Amresco) for 30 min at RT. HCASMCs were rinsed twice with water and observed the well-plates. For Alizarin Red staining, HCASMCs were fixed in 4% formalin for 15 min and washed twice with PBS. HCASMCs were stained with 2% Alizarin Red solution for 30 min at RT and rinsed twice with water and then well-plates were observed under the microscope.

(3) TNAP activity assay

TNAP activity was determined using Alkaline Phosphatase Activity Colorimetric Assay Kit (BioVision) according to the manufacturer's instructions.

(4) Calcium deposition assay

HCASMCs were washed with PBS and decalcified with 0.6 N HCl at 37 °C overnight. Calcium released from the cultured cell into the supernatant was measured by the Calcium Colorimetric Assay (BioVision), and total cellular protein was extracted by 0.1 N NaOH/0.1% SDS solution.

Figure S-4

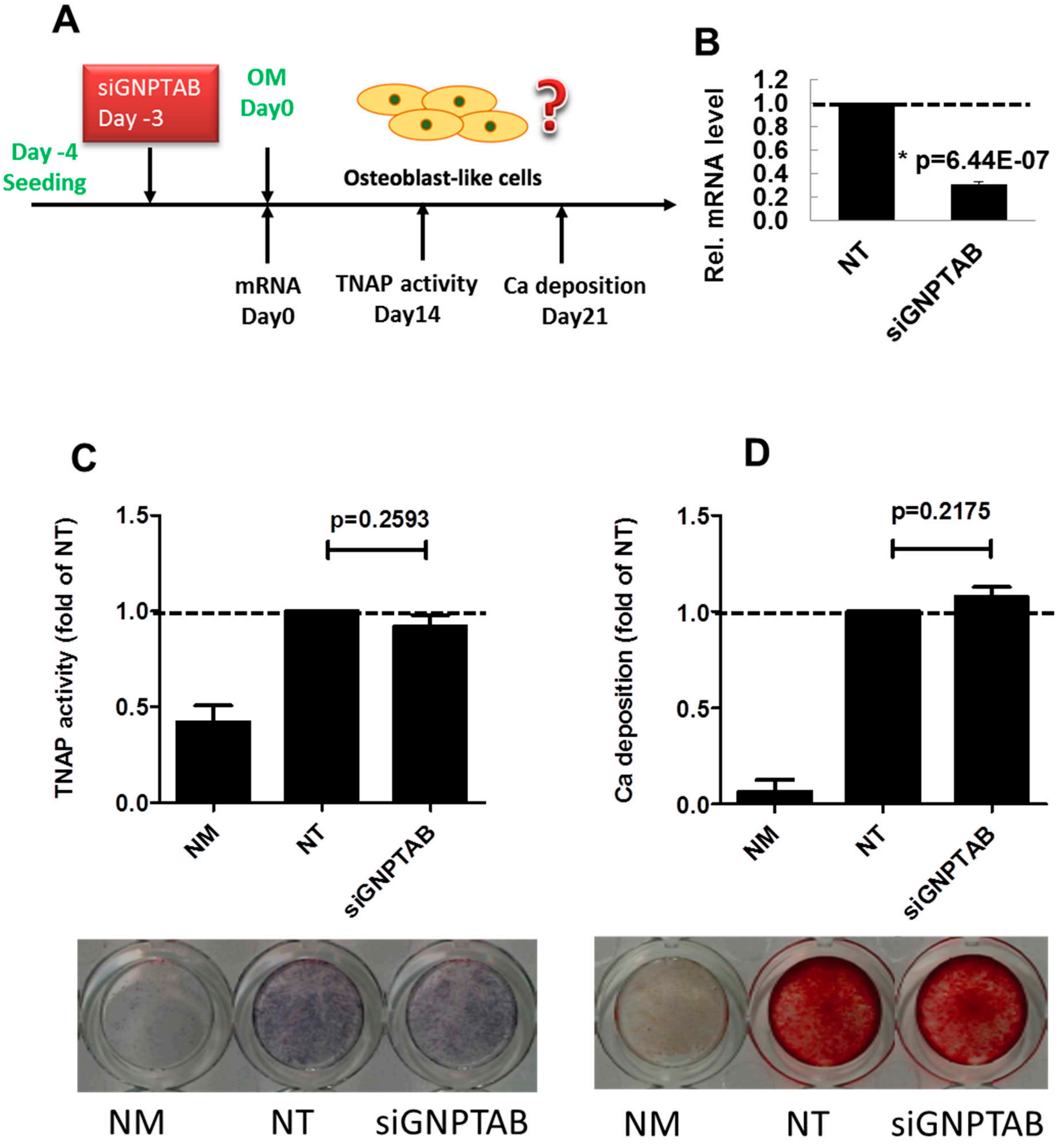

Figure S4. (**A**) Experimental design for siRNA experiments in SMC osteogenic model. (**B**) siRNA knockdown efficiency for siGNPTAB; **(C)** TNAP activity quantification and stain ($n = 3$); (**D**) Calcium deposition quantification and stain ($n = 3$).
